# Newspaper reporting of suicide in Nepal: Quality assessment against World Health Organization media guidelines

**DOI:** 10.1002/hsr2.547

**Published:** 2022-03-07

**Authors:** Rakesh Singh, Sharika Mahato, Seema Khadka, Pragyan Basnet, Kalendra Bista, Ritika Karki, S. M. Yasir Arafat

**Affiliations:** ^1^ Research Department Transcultural Psychosocial Organization (TPO) Nepal Kathmandu Nepal; ^2^ Visiting Faculty, Department of Community Medicine and Public Health KIST Medical College Lalitpur Nepal; ^3^ Monitoring, Evaluation and Research Department Plan International Nepal Lalitpur Nepal; ^4^ Helen Keller International Nepal Lalitpur Nepal; ^5^ School of Medicine Patan Academy of Health Sciences Lalitpur Nepal; ^6^ Department of Psychiatry Enam Medical College and Hospital Savar Bangladesh

**Keywords:** analysis, guideline adherence, Nepal, newspaper article, prevention and control, suicide, World Health Organization

## Abstract

**Background:**

Sensible media reporting of suicide is a population‐based suicide prevention strategy. However, the quality of media reporting of suicide has not been assessed in Nepal.

**Objectives:**

We aimed to assess the newspaper reporting status of suicide in Nepal with reference to World Health Organization (WHO) media guidelines for suicide reporting.

**Method:**

We retrospectively searched eight major newspapers in Nepal between January 2020 and May 2021 and assessed 167 news reports against WHO suicide reporting guidelines.

**Results:**

Potentially harmful characteristics were found to be reported in both the title and main text of the reports. About half of them mentioned sex (48.5%) and 38.3% mentioned the location of suicide in the title. Of the 167 reports, 74.3%, 95.2%, 34.7%, 92.2%, 98.8%, and 52.7% mentioned the name, sex, occupation, method of suicide, the location of suicide, and life events, respectively, in their main content. On the other hand, only 6% and 2.4% of reports mentioned linkage of suicides with mental illness and substance abuse, respectively. While lesser than 1% of reports narrated educative information regarding suicide prevention, none mentioned contact information for help‐seeking for the vulnerable.

**Conclusion:**

Newspaper reporting of suicide in Nepal poorly adheres to WHO guidelines, substantiated by the high presence of potentially harmful characteristics and negligible presence of potentially helpful characteristics.

## 1 INTRODUCTION

An estimate of 703,000 people died by suicide in 2019 accounting for 1.3% of all deaths worldwide.^1^ Among the suicides, 77% happened in low‐ and middle‐income countries in 2019 where prevention of suicide is under‐prioritized.^1^ The age‐standardized suicide rate was 10.2 per 100,000 in South‐East Asia, and 9.8 per 100,000 in Nepal in 2019 which was higher than the global average (9 per 100,000).^1^

Media plays a bidirectional role in the suicidal behavior of the general population, which is well‐known by the “Werther effect” and “Papageno effect.”^2^, ^3^, ^4^, ^5^, ^6^ Media reports can have potentially positive effects in suicidal prevention by disseminating the potentially helpful characteristics of media reporting, which has been circulated by World Health Organization (WHO).^5^ On the other hand, it can be harmful when it provides a simple, monocausal explanation, suggesting it as a solution to immediate triggers and, failing to explain the correlation with various proximal and distal risk factors.^7^, ^8^ Publishing of detailed descriptions of suicide methods can consequently result in imitation by the vulnerable people.^2^, ^9^, ^10^ Therefore, sensible media reporting of suicide is a population‐based suicide prevention strategy.^4^, ^5^ To minimize the inappropriate suicide reporting and promotion of sensible reporting, a guideline for media reporting of suicide has been developed by WHO and the *International Association for Suicide Prevention* (IASP).^5^ However, one recent review revealed that media rarely report the linkage of suicide with mental health, drug‐related issues, and usually do not include experts' opinions and factual statistics in South‐East Asian countires.^11^ Very little is known about the proper utilization of these recommended guidelines in Nepal. No report regarding assessment of the quality of media reporting on suicide in Nepal has been identified.^11^, ^12^ Hence, we aimed to explore the newspaper reporting status in Nepal against the WHO suicide reporting guidelines, which would be considered as a baseline study regarding quality analysis of media reporting on suicides in Nepal.

## 2 METHOD

### 2.1 Data collection

We searched the major printed national newspapers and major read online news portals in Nepal to identify the suicide news reports. The search was conducted by the first four authors. The newspapers and online news portals were selected based on daily nationwide circulation coverage and maximum hits of accessing the online sites, respectively. A retrospective search was done in six Nepali newspapers (two national printed and four online news portals) and two national English newspapers via their available online version. The two purposively selected English language newspapers were *The Himalayan Times* and *My Republica* and Nepali language newspapers and news portals were *Kantipur* and *Naya Patrika*, and *Onlinekhabar*, *Ratopati*, *Setopati*, and *Khabarhub*. News reports published between January 2020 and May 2021 were included in this analysis. We searched in *Google* using the term “suicide,” “Nepal,” names of the newspapers, “self‐harm,” “hanging self,” and “poisoning” in different combinations. The first author assessed the data quality by cross‐checking the reports and an acceptable level of inter‐rater reliability (Cohen's *κ* was above 0.90) was revealed.

### 2.2 Inclusion criteria

We included news of those specifically reported suicide in Nepal.

### 2.3 Exclusion criteria

We excluded news related to suicidal ideation, suicidal attempts, suicide bombing, opinion pieces on suicide, editor's view, and undecided suicide from the study. We did not include news published in languages other than English and Nepali and those published in local and regional newspapers.

### 2.4 Instrument, data extraction, and outcome variables

We identified and contrasted the variables against the WHO guidelines to assess adherence.^5^ We checked both potentially harmful and helpful characteristics as per the WHO guidelines. The instrument has already been used in various studies in different countries like Bangladesh, India, Indonesia, Myanmar, and Thailand.^13^, ^14^, ^15^, ^16^, ^17^ Potentially harmful characteristics include name, age, sex of the deceased, place, mode, attributed cause, pictorial presentation of suicide, life event related to suicide, and a suicide note. Whereas potentially helpful information targeting the prevention like help‐seeking behavior and services were extracted as outcome variables. We scrutinized the news reports and noted the outcome variables in MS Excel 2010 sheet on a “yes” and “no” basis. We also noted the basic details of news sources, including the name and language of the news outlet, link, and publication date for the online news reports. We presented the quality assessment of the news reports under three headings—content in the title, content in the body, and reporting education and information to the readers.

### 2.5 Statistical analysis

The Micro Soft Excel 2010 version and the Statistical Package for the Social Sciences (SPSS) were used for data analysis. We used frequency and percentage to describe the characteristics of media reports and performed *χ* ^2^ test and Fischer's exact test to determine the association between the type of media and quality of news reports and language of media and quality of news reports.

### 2.6 Ethical considerations

As the study analyzed publicly available information from online newspaper reports, no formal ethical clearance was sought for conducting the study.

## 3 RESULTS

### 3.1 Background characteristics of the suicide reports

Among the 167 suicide reports, 43.8% of the reports were from printed national newspapers and the rest 56.2% were from online national news portals (Table 1). Similarly, 87.4% of the suicide reports were from news media published in the national language (Nepali), and the remaining 12.6% were published in English (Table 1).

**Table 1:** Distribution of suicide reports as per language and type of media (*N* = 167)

Newspaper/online news portal	Type	Language	*N*	%
*Kantipur*	Print	Nepali	33	19.8
*Nayapatrika*	Print	Nepali	19	11.4
*The Himalayan Times*	Print	English	15	9.0
*My Republica*	Print	English	6	3.6
*Onlinekhabar*	Online	Nepali	37	22.2
*Ratopati*	Online	Nepali	20	12.0
*Setopati*	Online	Nepali	21	12.6
*Khabarhub*	Online	Nepali	16	9.6

### 3.2 Potentially harmful and helpful characteristics of Nepali media according to WHO suicide reporting guidelines

The potentially harmful characteristics were reported in both the title and main text of the suicide reports (Table 2). About half of them (48.5%) mentioned sex and 38.3% mentioned the location of suicide in the title. Of the 167 reports, 74.3%, 95.2%, 34.7%, 92.2%, 98.8%, and 52.7% narrated the name, gender, occupation, method, location of suicide, and life events, respectively, in the main content. Around 34% and 23% of the reports also mentioned the cause and steps of suicide in their main text, respectively.

**Table 2:** Nepali media adherence with WHO suicide reporting guidelines (*N* = 167)

Variables	*N*	%
*Potentially harmful characteristics*		
A. Mentioned information in the title		
Name	7	4.2
Age	4	2.4
Sex	81	48.5
Method of suicide	26	15.6
Location of suicide	64	38.3
Cause of suicide	29	17.4
Occupation	26	15.6
B. Mentioned information in the text		
Name	124	74.3
Age	122	73.1
Sex	159	95.2
Method of suicide	154	92.2
Location of suicide	165	98.8
Cause of suicide	57	34.1
Occupation	58	34.7
Steps of suicide	39	23.4
Life event	88	52.7
Suicide note	11	6.6
Citation from the suicide note	7	4.2
Suicide pact	4	2.4
Homicide	15	9.0
Effects on family or friends	5	3.0
Interview with the bereaved	16	9.6
Picture of deceased	8	4.8
*Potentially helpful characteristics*		
Suicide related to mental illness	10	6.0
Suicide related to drugs or alcohol use	4	2.4
Reporting warning signs	4	2.4
Expert opinion on suicide	2	1.2
Research findings on suicide	4	2.4
Statistics related to suicide	4	2.4
Mentioning preventive programs	1	0.6
Providing educative information	1	0.6
Reporting contacts for help‐seeking	0	0
Reporting the sources for further information	0	0

Abbreviation: WHO, World Health Organization.

Compared to harmful characteristics, helpful characteristics were found to be reported lesser in the suicide reports (Table 2). Only 6% and 2.4% were found to have reported linkage of suicides with mental illness and substance abuse, respectively. Similarly, only 2.4% mentioned warning signs, findings of national research on suicide and its statistics. While lesser than 1% narrated educative information regarding the prevention of suicide, none were found to have mentioned contact information for help‐seeking for individuals with suicidal thoughts.

### 3.3 Comparison of adherence with WHO suicide reporting guidelines between online news media and printed newspaper

While it was found that both forms of newspapers (online and printed) reported both harmful and helpful characteristics of suicide, no significant variation was noted between the forms of newspaper and quality of suicidal reports to conclude whether any of them had lesser or more harmful or helpful characteristics than its counterpart (Table 3).

**Table 3:** Comparison of adherence with WHO suicide reporting guidelines between online news media and printed newspaper (*N* = 167)

Variables	Online (*n* = 94), *f* (%)	Printed (*n* = 73), *f* (%)	*p* value
*Potentially harmful characteristics*			
A. Mentioned information in the title			
Name	3 (3.2)	4 (5.5)	0.7
Age	3 (3.2)	1 (1.4)	0.632
Sex	49 (52.1)	32 (43.8)	0.288
Method of suicide	12 (12.8)	14 (19.2)	0.257
Location of suicide	38 (40.4)	26 (35.6)	0.526
Cause of suicide	18 (19.1)	11 (17.4)	0.490
Occupation	13 (13.8)	13 (17.8)	0.482
B. Mentioned information in the text			
Name	66 (70.2)	58 (79.5)	0.176
Age	67 (71.3)	55 (75.3)	0.557
Sex	88 (93.6)	71 (97.3)	0.468
Method of suicide	85 (90.4)	69 (94.5)	0.327
Location of suicide	93 (98.9)	72 (98.6)	1
Cause of suicide	32 (34.0)	25 (34.2)	0.978
Occupation	28 (29.8)	30 (41.1)	0.128
Steps of suicide	22 (23.4)	17 (23.3)	0.986
Life event	48 (51.1)	40 (54.8)	0.632
Suicide note	3 (3.2)	8 (11.0)	0.060
Suicide note cited	3 (3.2)	4 (5.5)	0.700
Suicide pact	2 (2.1)	2 (2.7)	1
Homicide	7 (7.4)	8 (11.0)	0.587
Reporting effects to family or friends	3 (3.2)	2 (2.7)	1
Reporting the interview with the bereaved	8 (8.5)	8 (11.0)	0.594
Showing the picture of the victim	6 (6.4)	2 (2.7)	0.468
*Potentially helpful characteristics*			
Suicide related to mental illness	8 (8.5)	2 (2.7)	0.189
Suicide related to drugs or alcohol use	3 (3.2)	1 (1.4)	0.632
Reporting warning signs	2 (2.1)	2 (2.7)	1
Expert opinion on suicide	0	2 (2.7)	0.190
Research findings on suicide	3 (3.2)	1 (1.4)	0.632
Mentioning statistics related to suicide	3 (3.2)	1 (1.4)	0.632
Mentioning preventive programs or approach	0	1 (1.4)	0.437
Any educative information	0	1 (1.4)	0.437

Abbreviation: WHO, World Health Organization.

### 3.4 Comparison of adherence with WHO suicide reporting guidelines between online local language newspaper and English newspaper

While it was found that news media in both languages (English and Nepali) reported both harmful and helpful characteristics of suicide, there was no significant association between the language of news and quality of suicidal reports to conclude whether any of them had lesser or more harmful or helpful characteristics than its counterpart (Table 4).

**Table 4:** Comparison of adherence with WHO Suicide Reporting Guidelines between Nepali language media and English (*N* = 167)

Variables	English (*n* = 21), *f* (%)	Nepali (*n* = 146), *f* (%)	*p* value
*Potentially harmful characteristics*			
A. Mentioning information in the title			
Name	1 (5.0)	6 (4.1)	0.598
Age	0	4 (2.7)	1
Sex	8 (40.0)	73 (49.7)	0.417
Method of suicide	4 (20.0)	22 (15.0)	0.521
Location of suicide	14 (70.0)	50 (34.0)	0.002*
Cause of suicide	3 (15.0)	26 (17.7)	1
Occupation	3 (15.0)	23 (15.6)	1
B. Mentioning information in the text			
Name	12 (60)	112 (76.2)	0.120
Age	18 (90)	104 (70.7)	0.104
Gender	19 (95)	140 (95.2)	1
Method of suicide	19 (95)	135 (91.8)	1
Location of suicide	20 (100)	145 (98.6)	1.000
Cause of suicide	6 (30)	51 (34.7)	0.678
Occupation	6 (30)	52 (35.4)	0.636
Steps of suicide narrated	4 (20)	35 (23.8)	1
Life event	10 (50)	78 (53.1)	0.797
Suicide note	0	11 (7.5)	0.364
Suicide note cited	0	7 (4.8)	1
Suicide pact	1 (5.0)	3 (2.0)	0.403
Homicide	2 (10)	13 (8.8)	0.696
Reporting effects to family or friends	0	5 (3.4)	1
Reporting the interview with the bereaved	2 (10)	14 (9.5)	1
Showing the picture of the victim	0	8 (5.4)	0.598
*Potentially helpful characteristics*			
Suicide related to mental illness	0	10 (6.8)	0.610
Suicide related to drugs or alcohol use	0	4 (2.7)	1
Reporting warning signs	0	4 (2.7)	1
Expert opinion on suicide	0	2 (1.4)	
Research findings on suicide	0	4 (2.7)	1
Mentioning statistics on suicide	0	4 (2.7)	1
Mentioning preventive programs or approach	0	1 (0.7)	1
Any educative information	0	1 (0.7)	1

* *p* value significant at 0.05.

## 4 DISCUSSION

Although responsible media reporting is one of the effective population‐based suicide prevention strategies, it has not been explored previously in Nepal.^4^, ^5^, ^6^, ^11^, ^12^ We aimed to assess the quality of newspaper reports against WHO media guidelines for suicide reporting. We assessed 167 reports from eight prominent newspapers of Nepal between January 2020 and May 2021. Our study showed that media reporting suicide is usually nonadherent to WHO suicide reporting guidelines. The explicit mentioning of potentially harmful characteristics in both the title and main text of the reports was common practice, whereas reporting of potentially helpful characteristics was minimal. The narration of name, age, gender, method, and location of suicide in the main content is in more than one‐third of the suicidal reports. Educational information on suicide prevention was rarely mentioned and no information for help‐seeking behavior with suicidal thoughts was reported.

The findings of this study correspond to the reviews that assessed the quality of media reporting of suicide in South‐East Asia.^11^, ^12^ The low adherence to WHO guidelines and sensational presentation of suicides have been consistently found in other Asian counties also.^7^, ^15^, ^16^, ^17^, ^18^, ^19^, ^20^, ^21^, ^22^, ^23^, ^24^, ^25^, ^26^ One of the studies in India reported gender as the most common identity reported by the newspaper both in the title and main text.^24^ The minute reporting of cause and steps of suicide is found to be reported in this study, which can be potentially harmful to readers.^22^ Adherence to WHO suicide media reporting guidelines was also not practiced properly while reporting suicide reports in the newspaper and online media sources in China.^25^ These types of insensible practices can provoke suicidal behavior and can be imitated by vulnerable people; hence, controlling such practices is important.^27^, ^28^, ^29^

In contrast to potentially harmful characteristics, potentially helpful characteristics like providing hope to readers were less focused, which is comparable to the study findings from India, Bangladesh, Indonesia, Bhutan, Myanmar, and Thailand.^12^, ^15^, ^16^, ^17^, ^30^, ^31^ Apart from rarely mentioning educative information on suicide prevention, suicide linkage with mental illness, substance abuse, warning signs, and other potentially helpful reporting characteristics encouraged in the WHO guideline were not identified. Nothing was mentioned about supportive helpline/hotline information for the people with suicidal thoughts, which is similar to the study findings from Bangladesh.^13^ These can be due to the lack of awareness about media guidelines among the media personnel.^13^ The newspapers in India are also more focused on sensationalism and fail to incorporate educative materials on suicide prevention.^32^ This can be on account of lack of collaboration of media with mental health experts, poor mental health literacy, and absence of media monitoring.^22^ Hence, there is a dire need for collaboration of mental health professionals with media personnel for guidelines' implementation to prevent suicides.^18^, ^25^, ^32^ However, inadequacy in the number of mental health professionals in Nepal hinders such practices; thus, it is implausible as these professionals cannot contribute ample time to share their expertise with media professionals regularly.^22^ The WHO/IASP guideline further recommends using population‐level data, suicide research findings, mentioning a suicide hotline number, and supportive programs on suicide prevention; however, lack of population‐level data, few preventive and supportive programs, and paucity in research on suicide make the effective implementation of the guideline in Nepal a challenging task.^5^ Therefore, it is important to consider the recommendations of WHO/IASP guidelines in suicide reporting in Nepal.

This study reported no significant association between the forms of newspaper (online and printed) and quality of reports, which suggests that both forms of newspapers follow the same reporting pattern. Likewise, no significant association was found between the languages (English and Nepali) of news and the quality of reports. It is the same in the reports from Pakistan, where reporting was inappropriate in both Urdu and English language.^26^ However, other studies found that potentially helpful characteristics were reported more in English language newspapers compared to vernaculars.^30^, ^33^ One of the studies in India also reported the quality of suicide reporting in English newspapers, which focused on preventive aspects with lesser breaching in reporting compared to the regional language.^34^ These variations of the results in different studies suggest adhering to WHO suicide media reporting guidelines in both Nepali and English languages news portals in a similar fashion.

Psychiatric services could face a service burden after a celebrity suicide, which is portrayed with high negative characteristics in media reporting. Insensible media reporting may lead to misinformation among people that could lead to an increased burden of suicide or suicidal ideation/attempts. In the countries like Nepal, where the number of psychiatric professionals is very low, the rise in burden to deliver psychiatric services for increased cases attempting suicides would be more challenging.^35^ Also, tracing of psychiatric services should be regularly monitored while reporting the suicides in newspapers. As media plays an important role in disseminating information on suicide prevention information, it can be beneficial in decreasing suicide cases as well as preventing them. Therefore, raising awareness of the role of media in suicide prevention activities among media professionals is essential, and involving them in suicide prevention activities will be an additional asset. Besides, different challenges in the implementation of WHO guidelines on suicide reporting need to be considered while planning media engagement in such activities. There should have a regulatory mechanism for intense monitoring of the reports from media at regular intervals of time. The reporting guidelines should be modified and shaped as per the resources and needs of the country. Further, large‐scale studies assessing the quality of suicide media reporting in Nepal are recommended.

### 4.1 Strengths

This is one of the first studies conducted in Nepal to assess the quality of newspaper reporting suicide and can serve as a baseline study. This study can be helpful to media professionals to understand the current state and can encourage them in sensible media reporting, which can consequently help prevent suicide.

### 4.2 Limitations

There were some limitations in our study and the results should be generalized cautiously. This is a cross‐sectional study that included only eight printed and online news portals. Moreover, they were searched purposively and retrospectively. The information from newspapers may be insufficient, untrustworthy, and biased.^36^ We did not include other mass media, such as television and social media, which are the important source of news. We included only those articles which covered suicide deaths and excluded suicide attempts and reports discussing suicide topics.

## 5 CONCLUSION

Newspaper reporting was poor in adherence to WHO suicide reporting guidelines in Nepal. A detailed description of suicide cases was done frequently. Reporting of potentially harmful characteristics was common, while reporting of the helpful characteristics was minimal. There was negligible information about the supportive helpline for suicidal people. Awareness is needed and media professionals should be sensitized and engaged in appropriate and sensible media reporting of suicide. Strategic collaboration with media professionals, stakeholders, and mental health professionals should be designed to facilitate media professionals for their effective contribution to suicide prevention.

## CONFLICTS OF INTEREST

The authors declare no conflicts of interest.

## AUTHOR CONTRIBUTIONS

*Conceptualization*: Rakesh Singh, S. M. Yasir Arafat, and Sharika Mahato. *Data curation, validation, and visualization:* Rakesh Singh. *Formal analysis*: Rakesh Singh and Sharika Mahato. *Investigation*: Rakesh Singh, Pragyan Basnet, Kalendra Bista, and Ritika Karki. *Methodology*: Rakesh Singh, S. M. Yasir Arafat, and Sharika Mahato. *Project administration*: Rakesh Singh, Pragyan Basnet, Kalendra Bista, and Ritika Karki. *Supervision*: Rakesh Singh and S. M. Yasir Arafat. *Writing—original draft*: Rakesh Singh, Seema Khadka, and Sharika Mahato. *Writing—review and editing*: S. M. Yasir Arafat, Rakesh Singh, Sharika Mahato, Seema Khadka, Pragyan Basnet, Kalendra Bista, and Ritika Karki. All authors have read and approved the final version of the manuscript. The corresponding author had full access to all of the data in this study and takes complete responsibility for the integrity of the data and the accuracy of the data analysis.

## Data Availability

The data that support the findings of this study are available from the corresponding author upon reasonable request.
